# Expired Patents

**Published:** 1907-09

**Authors:** 


### EXPIRED PATENTS.

July 22, 1907.

432737. Artificial Tooth, C. H. Land, Detroit, Mich.

432909. Dental Mold, L. F. Seeger, Jr., Ahnapee, Wis.

July 29, 1907.

433185. Dental Vulcanizer, J. E. Quinn, Boston, Mass.

August 3, 1907.

433592. Combined Dental Mallet and Pliers, A. C. Corey, Council Grove, Kans.

August 12, 1907.

434317. Artificial Teeth, E. A. Bryant, Aspen, Colo.

August 19, 1907.

434697. Hand Piece Pulley Head for Dental Engines, A. W. Browne, Prince's Bay, N. Y.

434698. Attachment for Dental Engine Hand Pieces, A. W. Browne, Prince's Bay, N. Y.

434737. Dentist's Mixing Slab, L. Teal, Phila., Pa.

The above lists of patents are furnished by Davis & Davis, Solicitors of American and Foreign Patents. Washington, D. C., and St. Paul Building, New York City.

